# Initial Supplementary Dose of Dolutegravir in Second-Line Antiretroviral Therapy: A Noncomparative, Double-Blind, Randomized Placebo-Controlled Trial

**DOI:** 10.1093/cid/ciad023

**Published:** 2023-01-16

**Authors:** Ying Zhao, Rulan Griesel, Zaayid Omar, Bryony Simmons, Andrew Hill, Gert van Zyl, Claire Keene, Gary Maartens, Graeme Meintjes

**Affiliations:** Department of Medicine; Wellcome Centre for Infectious Diseases Research in Africa, Institute of Infectious Disease and Molecular Medicine; Wellcome Centre for Infectious Diseases Research in Africa, Institute of Infectious Disease and Molecular Medicine; Division of Clinical Pharmacology, Department of Medicine, University of Cape Town, South Africa; Department of Medicine; Wellcome Centre for Infectious Diseases Research in Africa, Institute of Infectious Disease and Molecular Medicine; LSE Health, London School of Economics and Political Science; Department of Pharmacology and Therapeutics, University of Liverpool, United Kingdom; Division of Medical Virology, Stellenbosch University, South Africa; Nuffield Department of Medicine, Health Systems Collaborative, Oxford Centre for Global Health Research, University of Oxford, United Kingdom; Wellcome Centre for Infectious Diseases Research in Africa, Institute of Infectious Disease and Molecular Medicine; Division of Clinical Pharmacology, Department of Medicine, University of Cape Town, South Africa; Department of Medicine; Wellcome Centre for Infectious Diseases Research in Africa, Institute of Infectious Disease and Molecular Medicine

**Keywords:** antiretroviral therapy, HIV, dolutegravir, efavirenz, second-line

## Abstract

**Background:**

Dolutegravir concentrations are reduced by efavirenz induction effect necessitating twice-daily dolutegravir dosing when coadministered. Efavirenz induction persists for several weeks after stopping, which could potentially select for dolutegravir resistance if switching occurred with unsuppressed human immunodeficiency virus type 1 (HIV-1) RNA levels and standard dolutegravir dosing. We evaluated the need for a lead-in supplementary dolutegravir dose in adults failing first-line tenofovir-emtricitabine-efavirenz (TEE).

**Methods:**

We conducted a randomized, double-blind, placebo-controlled, phase 2 trial in Khayelitsha, South Africa. Eligible patients had virologic failure (2 consecutive HIV-1 RNA ≥1000 copies/mL) on first-line TEE. Participants were randomly assigned (1:1) to switch to tenofovir-lamivudine-dolutegravir (TLD) with a supplementary 50 mg dolutegravir dose or placebo taken 12 hours later for 14 days. Primary outcome was proportion with HIV-1 RNA <50 copies/mL at week 24. This study was not powered to compare arms.

**Results:**

One hundred thirty participants were randomized (65 to each arm). Median baseline HIV-1 RNA was 4.0 log_10_ copies/mL and 76% had baseline resistance to both tenofovir and lamivudine. One participant died and 2 were lost to follow-up. At week 24, 55 of 64 (86% [95% confidence interval {CI}: 75%–93%]) in the supplementary dolutegravir arm and 53 of 65 (82% [95% CI: 70%–90%]) in the placebo arm had HIV-1 RNA <50 copies/mL. Grade 3 or 4 adverse events were similar in frequency between arms. None of 6 participants (3 in each arm) eligible for resistance testing by 24 weeks developed dolutegravir resistance.

**Conclusions:**

Our findings do not support the need for initial dolutegravir dose adjustment in patients switching to TLD who failed first-line TEE.

**Clinical Trials Registration:**

NCT03991013.

## Teaser

Among adults switching to second-line tenofovir-lamivudine-dolutegravir with unsuppressed HIV-1 RNA levels and substantial baseline nucleoside reverse transcriptase inhibitor resistance, a high proportion achieved virologic suppression.

Dolutegravir with 2 nucleoside reverse transcriptase inhibitors (NRTIs) is the World Health Organization (WHO)–recommended second-line antiretroviral therapy (ART) regimen for adults failing first-line regimens based on the nonnucleoside reverse transcriptase inhibitors (NNRTIs) nevirapine or efavirenz [1]. Based on evidence from the DAWNING study, which showed that dolutegravir was superior in safety and efficacy to lopinavir-ritonavir both administered with 2 NRTIs, at least 1 of which had to be fully active on resistance testing [2], current WHO guidelines recommend substituting tenofovir with zidovudine when switching to second-line ART, because the signature tenofovir resistance mutation K65R does not compromise zidovudine and low- and middle-income countries have limited access to resistance testing to guide selection of an optimized NRTI backbone [3].

Tenofovir is less toxic than zidovudine [4] and is dosed once rather than twice daily, which improves adherence. The NADIA study demonstrated that maintaining tenofovir at the transition to second-line dolutegravir-based or darunavir-based regimens was superior to switching to zidovudine in achieving virologic suppression at week 96 [5]. Emergent dolutegravir resistance has been reported in a small proportion of patients switching to second-line dolutegravir-based regimens in randomized trials [5, 6] and in a programmatic setting [7], which raises public health concerns as dolutegravir is recommended in first-line regimens in high-burden settings.

Efavirenz induces drug metabolizing enzymes (UGT1A1 and CYP3A4) and transporters (P-glycoprotein and breast cancer resistance protein), which decreases dolutegravir exposure [8]. Double-dose dolutegravir (50 mg twice daily) is recommended with efavirenz coadministration [9]. The induction effect of efavirenz persists after stopping and is largely resolved within 2 weeks [10]. Exposure to subtherapeutic dolutegravir concentrations in people switching from a tenofovir-emtricitabine-efavirenz (TEE) regimen with virologic failure to a tenofovir-lamivudine-dolutegravir (TLD) regimen could select for dolutegravir resistance, particularly when there is efavirenz and NRTI resistance present [10].

We previously conducted a prospective cohort study of recycled tenofovir and lamivudine with dolutegravir in second-line ART with a supplementary dolutegravir dose (50 mg twice daily) for 2 weeks to overcome efavirenz induction, and 85% achieved virologic suppression at week 24, despite 65% having resistance to both tenofovir and lamivudine at baseline [11]. It is unknown if a supplementary lead-in dose is necessary in people failing an efavirenz- and tenofovir-based regimen switching to second-line TLD. We therefore conducted a randomized placebo-controlled trial to assess the virologic efficacy of dolutegravir dose adjustment to overcome efavirenz induction when switching to TLD after virologic failure on TEE that is reported here.

## METHODS

### Study Design and Participants

ARTIST is a noncomparative, randomized, double-blind, placebo-controlled, phase 2, 48-week trial of second-line TLD with or without a lead-in supplementary dolutegravir dose in patients with virologic failure on first-line TEE. A detailed protocol describing our methods has been published [12]. We recruited patients from 3 primary care clinics in Khayelitsha, a large, periurban settlement in Cape Town, South Africa.

Eligible patients were adults (≥18 years old) with virologic failure (defined as 2 consecutive human immunodeficiency virus type 1 (HIV-1) RNA ≥1000 copies/mL 2–24 months apart) on a first-line regimen of tenofovir, emtricitabine (or lamivudine), and efavirenz. Exclusion criteria were CD4^+^ cell count <100 cells/µL; estimated glomerular filtration rate <50 mL/minute/1.73 m^2^; alanine aminotransferase >100 IU/L; total bilirubin more than twice the upper limit of normal; active opportunistic infection; active malignancy; pregnant or intention to become pregnant; breastfeeding; active psychiatric condition or substance abuse judged likely to impact adherence; and allergy or intolerance to one of the study drugs. Women of childbearing potential were required to receive effective contraception. On 14 July 2021, the study protocol was amended to include patients with CD4^+^ cell counts of between 50 and 100 cells/µL to facilitate recruitment.

### Randomization, Allocation Concealment, and Masking

Participants were randomly assigned (1:1) to a second-line regimen consisting of tenofovir (300 mg), lamivudine (300 mg), and dolutegravir (50 mg), given as a once-daily fixed-dose combination tablet, with an additional lead-in dose of dolutegravir (50 mg) or placebo taken 12 hours later for the first 14 days. An independent pharmacist generated the randomization sequence before trial commencement using block randomization (block size of 10). The study pharmacists used sequentially drawn individually sealed opaque envelopes to assign a treatment arm when dispensing medication. All participants and study staff involved in clinical care were blinded to treatment allocation.

### Procedures

Follow-up study visits with clinicians occurred at weeks 2, 4, 8, 12, 16, 20, and 24 (with a visit window of ±16 days at each visit except the week 24 visit, which had a window of ±6 weeks). Plasma HIV-1 RNA was measured at baseline and every visit from week 4 onward. If any HIV-1 RNA after week 12 was ≥50 copies/mL, or if there was <1 log_10_ decline in HIV-1 RNA from baseline, or if HIV-1 RNA was suppressed and subsequently rebound to ≥50 copies/mL, enhanced adherence counseling was performed, and HIV-1 RNA measurement was repeated after 2 weeks. If the repeat HIV-1 RNA was ≥500 copies/mL, a genotypic antiretroviral resistance test (GART) was performed. Baseline GART was performed retrospectively for all participants after completion of week 24 visits, on plasma samples stored at enrollment (with results not returned to clinicians). GART was performed at the National Health Laboratory Service Virology Laboratory at Tygerberg Hospital in Cape Town, South Africa, and drug susceptibility prediction was performed with the Stanford algorithm (version 9.1) [13].

CD4^+^ cell count was done at baseline and 24 weeks. We screened for insomnia using the Insomnia Severity Index (ISI) at baseline and every visit [14]. At baseline, 2, 4, 12, and 24 weeks, we screened for psychiatric symptoms using the Brief Symptoms Inventory Anxiety Subscale [15] and the Centre for Epidemiology Studies Depression Scale [16], and for neurocognitive impairment using the Simioni symptom questionnaire [17] and the cognitive assessment tool–rapid version [18].

We monitored adherence with tenofovir diphosphate (TFV-DP) concentrations at baseline, 12 weeks, and 24 weeks, using stored dried blood spot specimens. An indirect method for the quantification of TFV-DP in 50 mL human dried blood spots was adapted from a previously described method [19] and validated at the Division of Clinical Pharmacology at the University of Cape Town.

### Outcomes

The primary outcome was proportion with virologic suppression (defined as HIV-1 RNA <50 copies/mL) at week 24. We conducted a modified intention-to-treat (mITT) analysis according to the US Food and Drug Administration (FDA) snapshot approach, which defines failure as any 1 of HIV-1 RNA measurements ≥50 copies/mL, missing HIV-1 RNA within the window, intolerance or adverse event because of any study drug requiring switch, or loss to follow-up [20]. All participants who received at least 1 dose of the study drug were part of the analyzed cohort. Death from non-HIV and nondrug causes (as assessed by the study investigator) was not regarded as failure and the participant was excluded from the mITT population.

The secondary outcomes included virologic suppression at week 12 (with the same definition as for the primary outcome), proportion with HIV-1 RNA <400 copies/mL at weeks 12 and 24 (mITT; FDA Snapshot), time to virologic suppression, emergence of resistance mutations in participants meeting protocol-defined criteria for GART, including those with virologic failure (defined as 2 consecutive HIV-1 RNA ≥1000 copies/mL after week 12), and proportion developing grade 3 or 4 adverse events and serious adverse events. Adverse events were evaluated and graded at all study visits according to the Division of AIDS Table for Grading the Severity of Adult and Paediatric Adverse Events [21].

### Statistical Considerations

A sample size of 57 participants in each arm was estimated to produce a 95% confidence interval (CI) of 72%–92%, assuming the proportion achieving virologic suppression of 82% at week 24 (as achieved in the dolutegravir arm of the DAWNING study [2]). We increased the sample size to 65 in each arm to account for loss to follow-up. We calculated the proportion with virologic suppression with 95% CI for each arm. Time-to-event outcomes were analyzed using the Kaplan–Meier survival analysis. Prespecified sensitivity analyses of virologic suppression at weeks 12 and 24 were performed excluding individuals with evidence of poor adherence (TFV-DP concentration <350 fmol/punch); missing HIV-1 RNA within the window; loss to follow-up; and switching of study drug for reasons other than treatment failure. The study was not powered to demonstrate a statistical difference between the arms, and therefore, no formal efficacy comparison was conducted.

### Ethics Approval

Written informed consent was obtained from all participants. This study was approved by the Human Research Ethics Committee at the University of Cape Town (Ref 039/2019). We registered the study protocol on ClinicalTrials.gov (NCT03991013). A trial steering committee with independent members provided trial oversight and an independent data and safety committee reviewed interim safety data every 2 months.

## RESULTS

**Figure 1.**
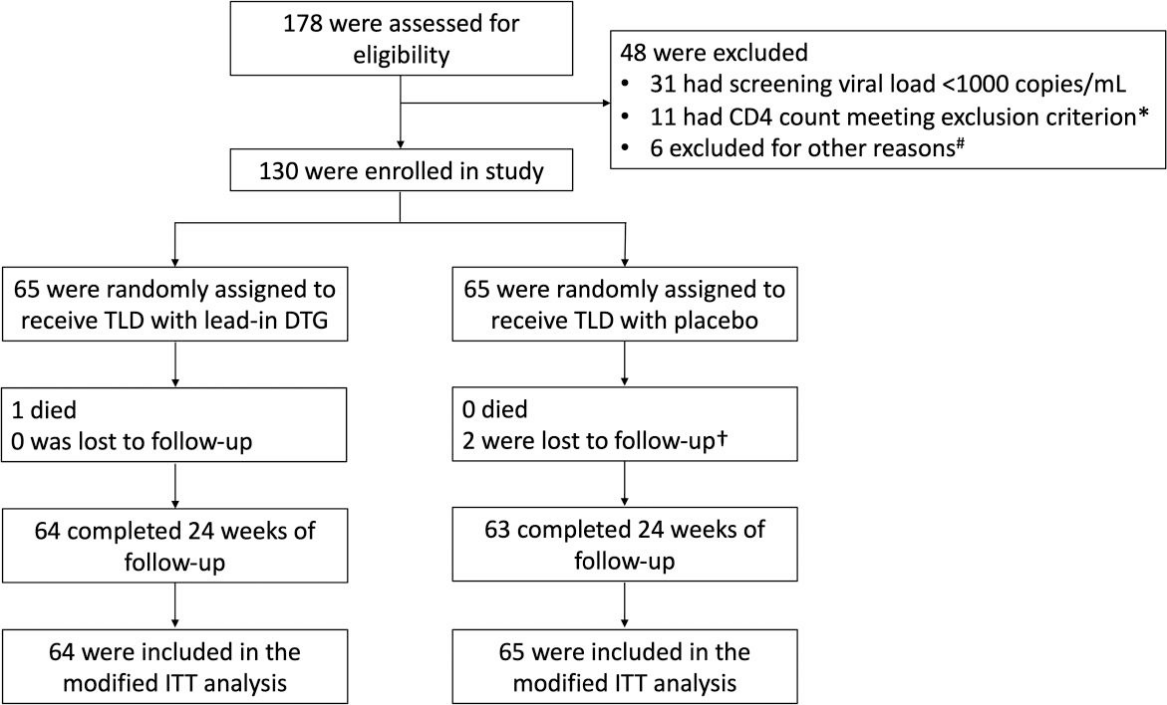
Eligibility assessment, randomization, treatment, and follow-up. *Eight patients had CD4 count <100 cells/µL and 3 had CD4 count <50 cells/µL. The protocol was amended to broaden the CD4 inclusion criterion from ≥100 to ≥50 cells/µL on 14 July 2021. ^#^Other reasons for exclusion accounted for <2% of patients screened (2 had positive pregnancy test, 1 had alanine aminotransferase >100 IU/L, 1 had active Kaposi sarcoma, 1 had a decrease of >2 log_10_ copies/mL between 2 most recent human immunodeficiency virus type 1 (HIV-1) RNA measurements, and 1 had significant cognitive impairment likely to impact adherence). †One participant met protocol-defined criteria for loss to follow-up but returned to the study at week 24 and had an HIV-1 RNA level ≥1000 copies/mL at week 24. Abbreviations: DTG, dolutegravir; ITT, intention-to-treat; TLD, tenofovir-lamivudine-dolutegravir.

Participants were recruited between 28 August 2020 and 10 November 2021. Of 178 adults screened, 130 participants were randomly assigned to receive lead-in supplementary dolutegravir (n = 65) or placebo (n = 65) (Figure 1). One participant died from severe coronavirus disease 2019, which was not considered drug-related or HIV-related, and therefore, 129 were included in the mITT analysis. Two participants were lost to follow-up before week 24. Baseline characteristics were well balanced between the randomized arms (Table 1). The majority (76%) of the 120 participants with successful baseline GART had mutations associated with at least low-level resistance to both tenofovir and lamivudine (Table 1).

**Table 1.** Baseline Characteristics

Variable	TLD + DTG (n = 65)	TLD + Placebo (n = 65)
Age, y, median (IQR)	38 (33–45)	39 (34–45)
Female sex, No. (%)	42 (65)	47 (72)
Weight, kg, median (IQR)	74 (62–87)	80 (63–96)
BMI, kg/m^2^, median (IQR)	28.4 (23.1–33.8)	30.1 (24.4–35.5)
CD4^+^ cell count, cells/µL, median (IQR)	246 (187–334)	250 (154–349)
HIV-1 RNA, log_10_ copies/mL, median (IQR)	4.12 (3.46–4.69)	4.01 (3.51–4.66)
Time receiving first-line ART, mo, median (IQR)	77 (45–112)	93 (57–132)
Previously received stavudine or zidovudine, No. (%)	4 (6)	12 (18)
NRTI genotypic resistance^a^, no./No. (%)^b^		
Two fully active NRTIs^c^	2/63 (3)	2/57 (4)
Resistance to 1 NRTI^a^	15/63 (24)	10/57 (18)
Tenofovir, not XTC	0/63 (0)	0/57 (0)
XTC, not tenofovir	15/63 (24)	10/57 (18)
Resistance to both NRTIs^a^	46/63 (73)	45/57 (79)
Efavirenz genotypic resistance^a^, no./No. (%)^c^	63/63 (100)	57/57 (100)
TFV-DP concentration, fmol/punch, median (IQR)	948 (725–1253)	1052 (733–1437)

Abbreviations: ART, antiretroviral therapy; BMI, body mass index; DTG, dolutegravir; HIV-1, human immunodeficiency virus type 1; IQR, interquartile range; NRTI, nucleoside reverse transcriptase inhibitor; TFV-DP, tenofovir diphosphate; TLD, tenofovir-lamivudine-dolutegravir; XTC, lamivudine or emtricitabine.

Resistance was classified with the Stanford algorithm, with a score of ≥15 indicating at least low-level resistance.

Denominators indicate the numbers of participants with available viral sequences.

Both NRTIs had a Stanford score <15 indicating susceptibility or only potential of low-level resistance.

Virologic outcomes by study arm at weeks 12 and 24 are shown in Table 2. At week 24, 55 (86% [95% CI: 75%–93%]) of 64 participants in the supplementary dolutegravir arm achieved virologic suppression (HIV-1 RNA <50 copies/mL) compared with 53 (82% [95% CI: 70%–90%]) of 65 participants in the placebo arm. Virologic outcomes at 24 weeks for the participants with FDA Snapshot failure were: in the supplementary dolutegravir arm 6 had HIV-1 RNA 50–99 copies/mL and 3 had HIV-1 RNA 100–999 copies/mL; in the placebo arm 4 had HIV-1 RNA 50–99 copies/mL, 2 had HIV-1 RNA 100–999 copies/mL, 4 had HIV-1 RNA ≥1000 copies/mL, 1 did not have an HIV-1 RNA test performed within the window, and 1 switched study drug following a tenofovir-related adverse event. Results of the sensitivity analyses were consistent with those of the mITT analyses (Table 2).

**Table 2.** Summary of Plasma Human Immunodeficiency Virus Type 1 RNA Outcomes

Variable	HIV-1 RNA <50 copies/mL, no./No. (% [95% CI])		HIV-1 RNA <400 copies/mL, no./No. (% [95% CI])	
	TLD + DTG (n = 65)	TLD + Placebo (n = 65)	TLD + DTG (n = 65)	TLD + Placebo (n = 65)
Week 24				
mITT analysis^a^	55/64 (86 [75–93])	53/65 (82 [70–90])	63/64 (98 [92–100])	59/65 (91 [81–97])
Sensitivity analysis^b^	55/64 (86 [75–93])	53/61 (87 [76–94])	63/64 (98 [92–100])	59/61 (97 [89–100])
Week 12				
mITT analysis^a^	53/64 (83 [71–91])	55/65 (85 [74–92])	61/64 (95 [87–99])	59/65 (91 [81–97])
Sensitivity analysis^b^	53/61 (87 [76–94])	55/61 (90 [80–96])	61/61 (100 [94–100])^c^	59/61 (97 [89–100])

Abbreviations: CI, confidence interval; DTG, dolutegravir; HIV-1, human immunodeficiency virus type 1; mITT, modified intention-to-treat; TLD, tenofovir-lamivudine-dolutegravir.

mITT analysis excludes those switching study drug for reasons of stopping contraception or desire to become pregnant, becoming pregnant, transfer out for nonclinical reasons, and death from non-HIV and nondrug causes.

Sensitivity analysis excludes those excluded from mITT analysis, as well as loss to follow-up, missing viral load within the window, switching study drug for reasons other than treatment failure, and evidence of poor adherence (tenofovir diphosphate <350 fmol/punch).

One-sided 97.5% CI when 0 or 100% were successful.

**Figure 2.**
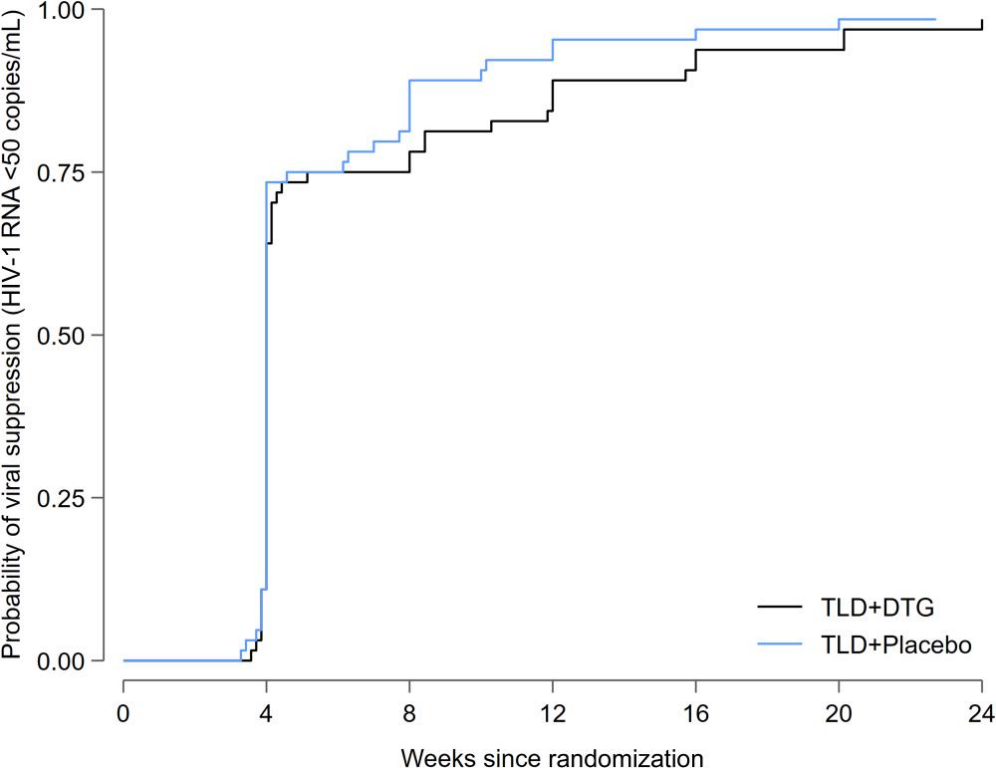
Kaplan-Meier for time to virological suppression (human immunodeficiency virus type 1 RNA <50 copies/mL). Abbreviations: DTG, dolutegravir; HIV-1, human immunodeficiency virus type 1; TLD, tenofovir-lamivudine-dolutegravir.

In the subgroup with resistance to both tenofovir and lamivudine at baseline, virologic suppression at week 24 was achieved by 39 of 45 (87% [95% CI: 73%–95%]) participants in the supplementary dolutegravir arm and 36 of 45 (80% [95% CI: 65%–90%]) participants in the placebo arm. Time to virologic suppression by arm is shown in Figure 2. Median time to virologic suppression was 4.0 weeks (interquartile range [IQR], 4.0–5.1 weeks) in the supplementary dolutegravir arm and 4.0 weeks (IQR, 4.0–6.1 weeks) in the placebo arm.

Six participants (3 in each arm) met protocol-defined criteria for GART by week 24; no participants developed dolutegravir resistance or acquired new resistance mutations to tenofovir or lamivudine. One participant in the supplementary dolutegravir arm developed virologic failure at week 16; GART detected no integrase resistance mutations and the participant reported poor adherence (corroborated by TFV-DP concentration <350 fmol/punch at week 12). There were 19 participants with HIV-1 RNA ≥50 copies/mL at week 24; 15 of 19 (79%) later resuppressed HIV-1 RNA <50 copies/mL with enhanced adherence counseling.

**Figure 3.**
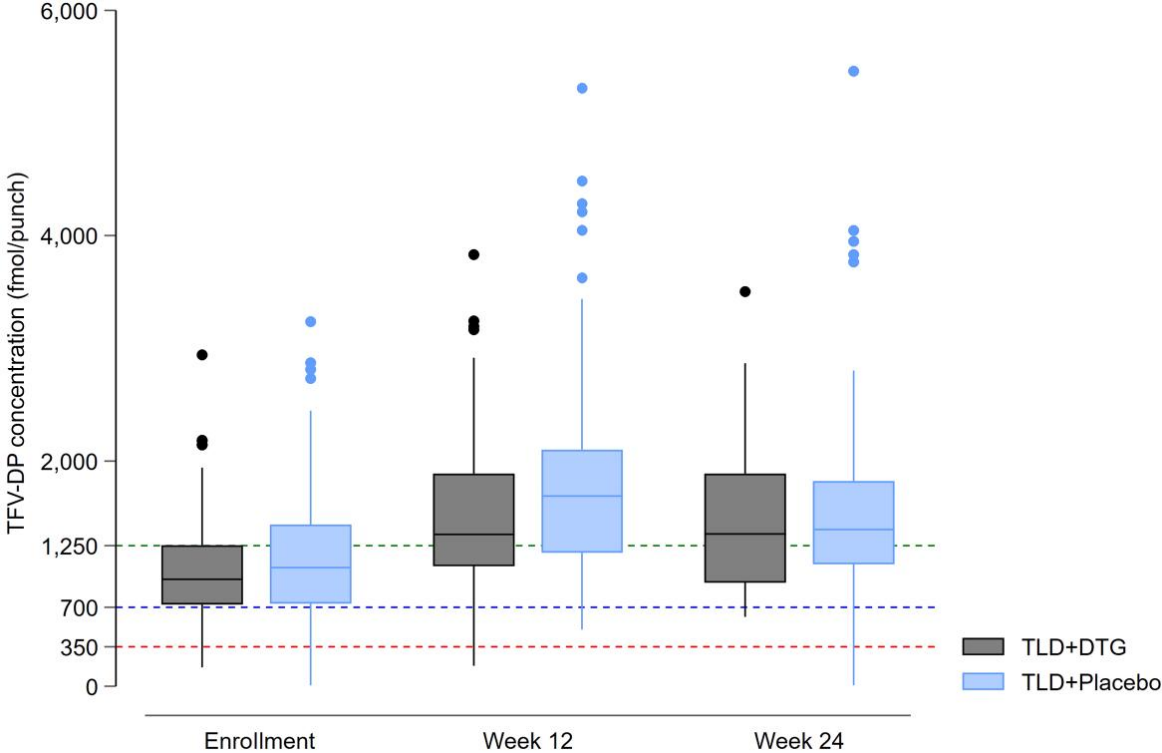
Tenofovir diphosphate (TFV-DP) concentrations at baseline, 12 weeks, and 24 weeks (boxes indicate interquartile range, horizontal solid lines are medians, vertical lines are ranges, and solid circles are outliers). Dotted lines refer to TFV-DP concentrations categorized using the threshold defined by Anderson et al [22] as <350 fmol/punch (men: <1.2 doses per week and women: <0.6 doses per week), 350–700 fmol/punch (men: 1.2–3.2 doses per week and women: 0.6–2.0 doses per week), 700–1250 fmol/punch (men: 3.2–6 doses per week and women: 2.0–5.3 doses per week), and >1250 fmol/punch (men: >6 doses per week and women: >5.3 doses per week). Abbreviations: DTG, dolutegravir; TFV-DP, tenofovir diphosphate; TLD, tenofovir-lamivudine-dolutegravir.

Median increase in CD4^+^ cell count at week 24 was 88 cells/µL (IQR, 45–138 cells/µL) in the supplementary dolutegravir arm and 75 cells/µL (IQR, 18–127 cells/µL) in the placebo arm. Median increase in weight was 1.9 kg (IQR, −0.9 to 4.8 kg) over 24 weeks, to 78.1 kg in the supplementary dolutegravir arm, versus 1.4 kg (IQR, −1.7 to 4.9 kg) to 82.0 kg in the placebo arm. Median TFV-DP concentrations were similar between arms at baseline, 12 weeks, and 24 weeks (Figure 3). Complete adherence to the additional dose of dolutegravir/placebo (assessed as no missed dose by returned pill counts) was 77% in the supplementary dolutegravir arm and 70% in the placebo arm.

**Figure 4.**
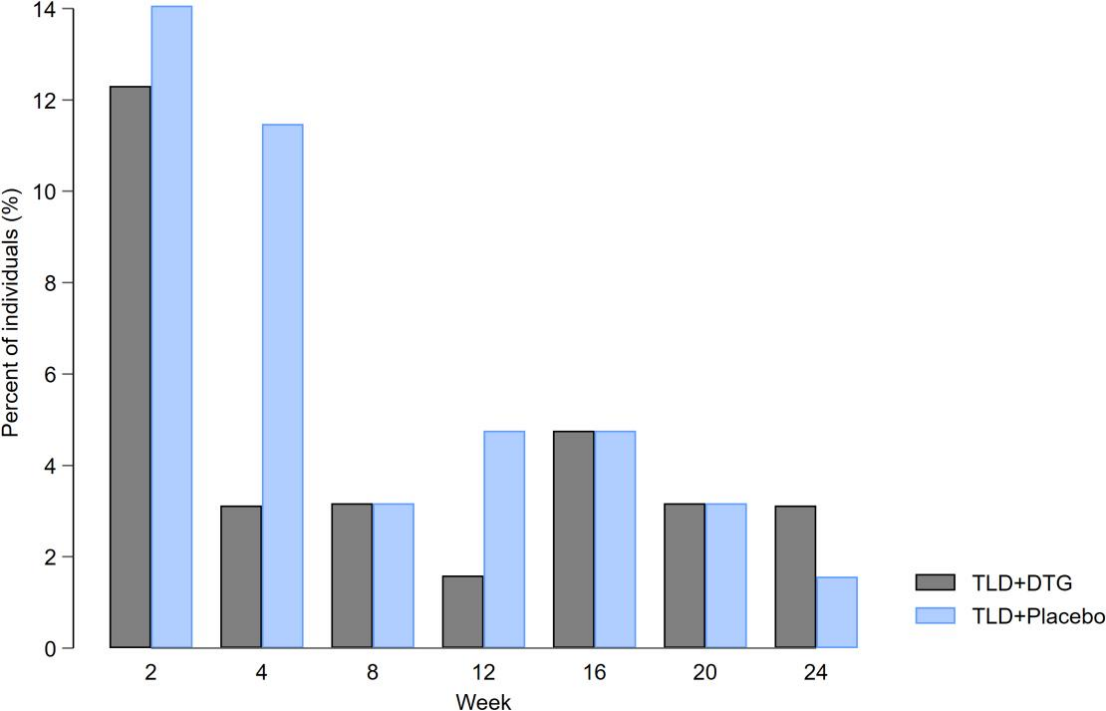
Proportion of participants with a ≥1-unit increase in the Insomnia Severity Index (ISI) and reported insomnia as an adverse event. Sleep was evaluated using the ISI, a 7-item tool with a total score ranging from 0 to 28. A higher score indicates a worse performance. Numerator is the number of individuals who reported insomnia with at least a 1-unit increase in ISI score from baseline (week 0). Denominator is the number of individuals with an ISI score at each week for all individuals with a baseline result. Abbreviations: DTG, dolutegravir; TLD, tenofovir-lamivudine-dolutegravir.

Grade 3 or 4 adverse events and serious adverse events were uncommon (Table 3). At 2 weeks, 8 (12%) participants in the supplementary dolutegravir arm and 9 (14%) participants in the placebo arm reported insomnia as an adverse event and had a ≥1 ISI score change from baseline, with rapid attenuation of symptoms and ISI scores over time (Figure 4). Two (3%) participants in the supplementary dolutegravir arm and 8 (13%) participants in the placebo arm had ISI scores above diagnostic threshold for clinically significant insomnia (ISI scores ≥8) [14] over 24 weeks; none resulted in the discontinuation of therapy. Emergence of anxiety, depression, and cognitive complaints were low across the arms. One grade 3 psychiatric adverse event (psychosis) occurred in the supplementary dolutegravir arm, which was not considered treatment related. One participant developed tuberculosis at week 8; dolutegravir dose was increased to 50 mg twice daily until 2 weeks after completing rifampicin.

**Table 3.** Number (%) of Participants With Adverse Events

Adverse Event	TLD + DTG (n = 65)	TLD + Placebo (n = 65)
Any grade 3–4 AE, SAE, or death	3 (5)	5 (8)
Mortality (all-cause)	1 (2)^a^	0
Any SAE^b^	2 (3)	0
Any grade 3–4 AE	2 (3)	5 (8)
Grade 3^c^	2 (3)	5 (8)
Grade 4	0	0
AE leading to discontinuation of study drug	0	1 (2)^d^

Abbreviations: AE, adverse event; DTG, dolutegravir; SAE, serious adverse event; TLD, tenofovir-lamivudine-dolutegravir.

The death in the DTG arm was caused by severe coronavirus disease 2019 (COVID-19).

The SAEs in the DTG arm were severe COVID-19 leading to death, and acute psychosis, which was considered unrelated to the study drug and resolved without discontinuation of the study drug.

The grade 3 AEs in the DTG arm were acute psychosis and incident pulmonary tuberculosis. The grade 3 AEs in the placebo arm were raised random glucose (experienced by 2 participants), acute kidney injury (experienced by 2 participants), and headache.

One participant in the placebo arm switched from tenofovir to zidovudine after developing a creatinine elevation.

## DISCUSSION

In our study, second-line TLD produced acceptable rates of virologic suppression at 24 weeks in adults with virologic failure on first-line TEE, with and without a lead-in supplementary dolutegravir dose. No emergent dolutegravir resistance occurred over 24 weeks among our cohort of patients who switched with unsuppressed HIV-1 RNA levels, despite the majority having resistance to both tenofovir and lamivudine at baseline. Our findings strengthen the evidence base for recycling tenofovir and lamivudine with dolutegravir in second-line ART in resource-limited settings.

Our results are consistent with those from previous randomized controlled trials assessing the efficacy of dolutegravir in second-line ART [2, 5, 23]. In the NADIA study, 92% of participants who maintained tenofovir in second-line regimens achieved HIV-1 RNA <400 copies/mL at week 48 [24]. At week 96, recycling tenofovir was superior to switching to zidovudine in NADIA [5]. In the VISEND study, 83% of participants randomized to TLD with preswitch HIV-1 RNA >1000 copies/mL achieved HIV-1 RNA <1000 copies/mL at week 48 [23]. Most of our participants (76%) had resistance to both NRTIs at baseline and the proportion achieving virologic suppression in this subgroup was similar to that achieved by the same subgroup in NADIA [24]. It is well established that the modest effect of NRTIs on reducing viral fitness in the presence of NRTI resistance mutations is both necessary and sufficient to achieve virologic suppression in combination with a protease inhibitor [25]; this appears to also be true with dolutegravir. The proportion of patients who had virologic suppression while taking a dolutegravir-based second-line regimen was similar to that among patients taking a well-tolerated protease inhibitor–based second-line regimen [5, 23].

A previous study conducted among healthy, HIV-negative volunteers showed that efavirenz reduced dolutegravir trough concentrations by 75% when coadministered, but that doubling the dolutegravir dose mitigated the drug–drug interaction [8]. In a company-sponsored study, dolutegravir and efavirenz concentrations did not simultaneously fall below their respective clinical target concentrations after switching from efavirenz to dolutegravir [26]. Based on that study, dolutegravir dose adjustment is not recommended for patients who switch with suppressed HIV-1 RNA levels. However, there has been concern that among patients with efavirenz and NRTI resistance who switch with unsuppressed HIV-1 RNA levels, dolutegravir resistance may be selected in some individuals. Our results suggest that initial dolutegravir dose adjustment is not required when switching from efavirenz to dolutegravir among viremic individuals, and therefore, the pill burden and cost of an additional (non-fixed-dose combination) dose of dolutegravir when transitioning to second-line can be avoided.

Emergent dolutegravir resistance has been documented infrequently in second-line ART (4% at 96 weeks in NADIA, and 2% at 159 weeks in DAWNING) [5, 6]. In a prospective cohort study of 1892 Malawian patients who were transitioned from NNRTI-based first-line to TLD without preswitch HIV-1 RNA testing, 2 cases of dolutegravir resistance were detected at 6 months; both patients were viremic at switch and received dolutegravir with no predicted active NRTIs [7]. Intermittent adherence, drug–drug interactions, high baseline HIV-1 RNA levels, and active opportunistic infections are risk factors associated with emergent dolutegravir resistance [27]. The lack of dolutegravir resistance in our cohort over 24 weeks may be attributed to optimal adherence in the majority supported with intensive adherence counseling, the exclusion of patients with active AIDS-defining conditions and contraindicated drug–drug interactions, and the relatively low median HIV-1 RNA levels at study entry. Further research into the risks associated with and mechanisms underlying integrase mutation selection is needed to better predict the development of dolutegravir resistance and determine the clinical consequence of dolutegravir resistance, particularly when combined with preexisting NRTI resistance.

In ARTIST, we observed a substantial incidence of insomnia (13%) reported by participants at week 2 with no discernible difference in the proportion between arms; the majority of insomnia complaints were mild and did not reach clinical significance based on ISI scores, and 76% resolved by week 4. The incidence of insomnia we found is twice that reported in a meta-analysis of trial participants randomized to dolutegravir [28]. However, previous studies have not evaluated insomnia in the first 2 weeks after initiating dolutegravir, and early events that resolved by 4 weeks may have been missed. Our findings highlight the need for further research to assess neuropsychiatric adverse events in larger patient populations that should be part of pharmacovigilance initiatives with the current transition to dolutegravir-based regimens.

Our study has limitations. First, our study was not powered to formally assess differences between arms. Although the sample size did not allow for a statistically powered comparison, our study was designed to rapidly generate data to supplement the findings from NADIA [5] and the ongoing D²EFT study (estimated date of completion in July 2023) [29] to inform clinical care for patients transitioning to dolutegravir-based regimens in resource-limited settings. Second, our primary virologic outcome was at 24 weeks; the development of dolutegravir resistance is often delayed [5, 6]. Dolutegravir resistance may be detected with longer-term follow-up. Third, all our participants received frequent HIV-1 RNA monitoring and intensive adherence counseling, and we excluded patients with active AIDS-defining conditions. The results may not be generalizable to patients receiving treatment under programmatic conditions where HIV-1 RNA monitoring is infrequent.

## CONCLUSIONS

The ARTIST study provides additional evidence that maintaining tenofovir and lamivudine with dolutegravir is effective and well tolerated in second-line ART. Our results provide evidence over 24 weeks that patients with unsuppressed HIV-1 RNA levels on first-line TEE can safely switch to second-line TLD without a lead-in supplementary dolutegravir dose to overcome efavirenz induction—however, it is possible that resistance will emerge at later timepoints.

## Notes

***Acknowledgments*.** The authors wish to acknowledge clinical, administrative, and managerial staff of the Wellcome Centre for Infectious Diseases Research in Africa: Zimasa Gcwabe, Kaneez Sayed, Sydney Ncoko, Mathilda Nduna, Sihle Nqina, Zimkhitha Asare, Amanda Jackson, Olina Ngwenya, Rene Goliath, Meagan McMaster, and Charlotte Schutz, as well as Tasanya Chinsamy, Kirsten Arendse, Tali Cassidy, and Eric Goemaere from Médecins Sans Frontières. We thank the clinical and managerial staff at Ubuntu, Michael Mapongwana, and Nolungile Community Health Centres for assisting with recruitment. We thank the data and safety monitoring committee members for reviewing interim safety data: Joseph Jarvis, David Meya, Daniela Garone, and Catherine Orrell, as well as independent members of the trial steering committee for providing trial oversight: Francois Venter and Richard Kaplan. We thank the ARTIST study participants for their commitment to the trial.

***Disclaimer.*** The funders had no role in the study design, data collection, data analysis, data interpretation, or writing of this report. The opinions, findings, and conclusions expressed in this manuscript reflect those of the authors alone. The corresponding author had full access to all the data in the study and had final responsibility for the decision to submit for publication. For the purpose of open access, the author has applied a CC BY public copyright licence to any author-accepted manuscript version arising from this submission.

***Financial support*.** This work was supported by an investigator award from the Wellcome Trust (212265/Z/18/Z) and the Médecins Sans Frontières Khayelitsha Project. The Wellcome Centre for Infectious Diseases Research in Africa was supported with core funding from the Wellcome Trust (203135/Z/16/Z). G. Me. was supported by the Wellcome Trust (214321/B/18/Z) and the South African Research Chairs Initiative of the Department of Science and Technology and National Research Foundation of South Africa (grant number 64787). The University of Cape Town Clinical Pharmacology Laboratory was supported by the National Institute of Allergy and Infectious Diseases of the National Institutes of Health (award numbers UM1 AI068634, UM1 AI068636, and UM1 AI106701). B. S. also reports support for this work from University of Cape Town, Cape Town, South Africa (consultancy payment to author for statistical components of the manuscript).
